# Beliefs of Health Care Providers, Lay Health Care Providers and Lay Persons in Nigeria Regarding Hypertension. A Systematic Mixed Studies Review

**DOI:** 10.1371/journal.pone.0154287

**Published:** 2016-05-05

**Authors:** James Tosin Akinlua, Richard Meakin, Philip Fadahunsi, Nick Freemantle

**Affiliations:** 1 Department of Primary Care and Population Health, University College London Medical School (Royal Free Campus), Rowland Hill street, London, NW3 2PF, United Kingdom; 2 Department of Primary Care and Public health, School of Public Health, Faculty of Medicine, Imperial College London, Charing Cross Campus, Reynolds building, St. Dunstan road, London, W6 8RP, United Kingdom; Western Sydney University, AUSTRALIA

## Abstract

**Background:**

Hypertension is a major health risk factor for mortality globally, resulting in about 13% of deaths worldwide. In Nigeria, the high burden of hypertension remains an issue for urgent attention. The control of hypertension, among other factors, is strongly determined by personal beliefs about the illness and recommended treatment.

**Objective:**

The aim of this review is to systematically synthesize available data from all types of studies on beliefs of the Nigerian populace about hypertension

**Methods:**

We searched the following electronic databases; Medline, EMBase, PsycInfo, AMED from their inception till date for all relevant articles. A modified Kleinman’s explanatory model for hypertension was used as a framework for extraction of data on beliefs about hypertension.

**Results:**

The search yielded a total of 3,794 hits from which 16 relevant studies (2 qualitative, 11 quantitative and 3 mixed methods studies) met the inclusion criteria for the review. Overall, most health care providers (HCPs) believe that stress is a major cause of hypertension. Furthermore, reported cut-off point for uncomplicated hypertension differed widely among HCPs. Lay Health Care Providers such as Patent Medicine Vendors’ beliefs about hypertension seem to be relatively similar to health care professionals in areas of risk factors for hypertension, course of hypertension and methods of treatment. Among Lay persons, misconception about hypertension was quite high. Although some Nigerians believed that life style habits such as alcohol intake, exercise levels, cigarette smoking were risk factors for developing hypertension, there was discordance between belief and practice of control of risk factors. However, beliefs across numerous ethnic groups and settings (urban/rural) in Nigeria have not been explored.

**Conclusion:**

In order to achieve control of hypertension in Nigeria, interventions should be informed, among other factors, by adequate knowledge of beliefs regarding hypertension across the numerous ethnic groups in Nigeria, settings (rural/urban), age and sex.

## Introduction

Nearly 1 billion people were reported to have hypertension in the year 2000 with over half of this number resident in developing countries and this number is projected to increase by about 5% by the year 2025 in the same proportions [1, 2, 3]. Nigeria with a population of over 170million people forms a substantial part of this burden in sub Saharan Africa. However, the degree of control of blood pressure in Sub-Saharan Africa including Nigeria is far less than that obtained in developed nations [4, 5].

Although there are different barriers that hinder hypertension control at individual, institutional and health care provider levels, one key individual related factor that determines control of hypertension is adherence to recommended therapy [6,7].

Adherence/Non-adherence to recommended treatment is dependent on socio-cultural, economic and individual factors such as pre-existing beliefs about the illness and available treatment modalities [8, 9, 10,11]. Out of all of these factors, personal beliefs about illness and treatment seem to be most important factor when change of behaviour is required [12,13].

Important beliefs which predict individuals’ attitude and behaviour about treatment could be potentially explained conveniently using the Necessity-Concerns Framework which postulates that adherence/non-adherence to medications is dependent on 2 key categories of common-sense evaluations: Necessity beliefs i.e. personal opinion about the need for treatment and concerns about potential unpleasant repercussions [9, 14, 15,16].

Furthermore, individual beliefs about illnesses and reactions to health threats can be understood from different perspectives. The anthropological viewpoint is particularly useful in the context of comparing, analysing and explaining different cultures.

Of note is the Kleinman’s anthropological explanatory model (EM) which was used to explain the differences between lay health beliefs and biomedical beliefs of health care providers [17, 18, 19]. Agreement between the EM of the health care provider and sick person has been reported to have positive impact on sick person outcomes. On the other hand, disagreement between EMs may result in negative outcomes such as non-adherence to recommended treatment methods [13].

Research studies that have applied this model have suggested that people’s belief about hypertension differed from the orthodox bio-medical perspective [17, 18, 19, 20]. Furthermore, studies have shown different beliefs about hypertension among black people from different ethno-cultural backgrounds [21, 22]. But, information about the shared and divergent beliefs of Nigerians who may belong to one of over 250 ethnic groups on hypertension is very limited.

Therefore, this paper seeks to address this gap in the literature by providing a systematic literature review of beliefs of the population in Nigeria regarding hypertension.

### Review Question

What is known about beliefs of the population in Nigeria regarding hypertension?

## Objectives

The aim of this review is to review systematically the literature to identify and explore beliefs of the Nigerian populace (including health care providers) about hypertension. We included qualitative studies to gain in-depth understanding of people’s perception about hypertension and quantitative studies to provide information on prevalence of various concepts and their clinical relevance. In addition to primary qualitative and quantitative studies, mixed methods studies that attempted to ascertain beliefs of Nigerians about hypertension were also included.

## Methods

### Protocol

The Methods used in this review were determined in advance and documented in a protocol.

### Explanatory Model theoretical framework

Beliefs in this review were defined using the Kleinman’s explanatory model (EM) of illness as a template to organise studies’ findings into different EM categories.

According to Kleinman, explanatory model of an illness refer to “the notions (beliefs) about an episode of sickness and its treatment that are employed by all those engaged in the clinical process” (p.105) [23]^.^ A lay person’s explanatory model of illness consist of the following items: a) what is the cause of my illness? (b) Why did I fall sick at this particular time? (c) How does this illness operate in my body system? (d) How will this illness affect me, what will the illness do to me? (e) How should this illness be treated?

On the other hand, a health care provider’s explanatory model include: the cause of illness (aetiology); time and mode of onset; patho-physiology (or mechanism) of disease; course of the disease including symptoms and signs and recommended treatment [23, 24, 25, 26].

Moreover, Kleinman combined the lay and biomedical explanatory models to derive a generalised structure for explanatory models which allows lay and biomedical models to be categorised in a single structure namely: “cause/aetiology”, “course of illness”, “patho-physiology”, “symptoms” and “treatment” [24].

However, in this review, “definition” was added to the explanatory model for hypertension.

In this review, Lay Persons shall refer to persons who are known hypertensive patients or at risk of becoming hypertensive. Complementary and alternative medicine healthcare providers (CAM healers) and patent medicine vendors (PMV) will be collectively known as Lay Health Care Providers. Further, the term Health Care Provider shall refer to a physician, nurse, community health extension worker or pharmacist who has been trained in the biomedical perspective.

### Definitions of EM categories and assumptions

Based on the modified Kleinman’s EM of illness described above, beliefs or perceptions about 6 EM categories (“definition”, “cause”, “course”, “patho-physiology”, “symptoms” and “treatment”) were extracted from studies included in this review. The following definitions and assumptions apply for EM categories included in this review.

**Definition**: shall refer to what hypertension is? **Cause:** shall refer to beliefs or perceptions about the cause of hypertension **Course**: shall refer to beliefs about how the illness evolves or operate in the body and complications arising from non-treatment of disease. As such, it is assumed that beliefs about biomedical complications cited as motivation for adherence to treatment recommendations could be regarded as perception of the course of the disease. **Pathophysiology**: shall refer to biomedical explanation of the disordered activities that occurs in a disease or injury. **Symptoms**: shall refer to beliefs about what the illness will do to one’s body. **Treatment**: shall refer to beliefs or perceptions about effective treatment modalities for hypertension. It also includes beliefs about whether hypertension could be cured or not.

## Information Sources and Search Strategy

A robust search strategy was developed to identify qualitative and quantitative studies. We searched the following electronic databases; Medline, EMBase, PsycInfo, AMED from their inception till date (week 2 July 2015) for all relevant articles. We also hand searched reference lists to identify other important articles that the electronic database search might have missed. Specifically, qualitative studies were identified using proven sensitive methodological terms for qualitative research [27]. Details of search results are presented in S1 and S2 Tables. No limits were applied with regards to language, year of publication, age, sex or groups of people. But the limit non-human was applied to exclude all articles conducted on non-humans.

## Eligibility Criteria and Study Selection

**Fig 1:**
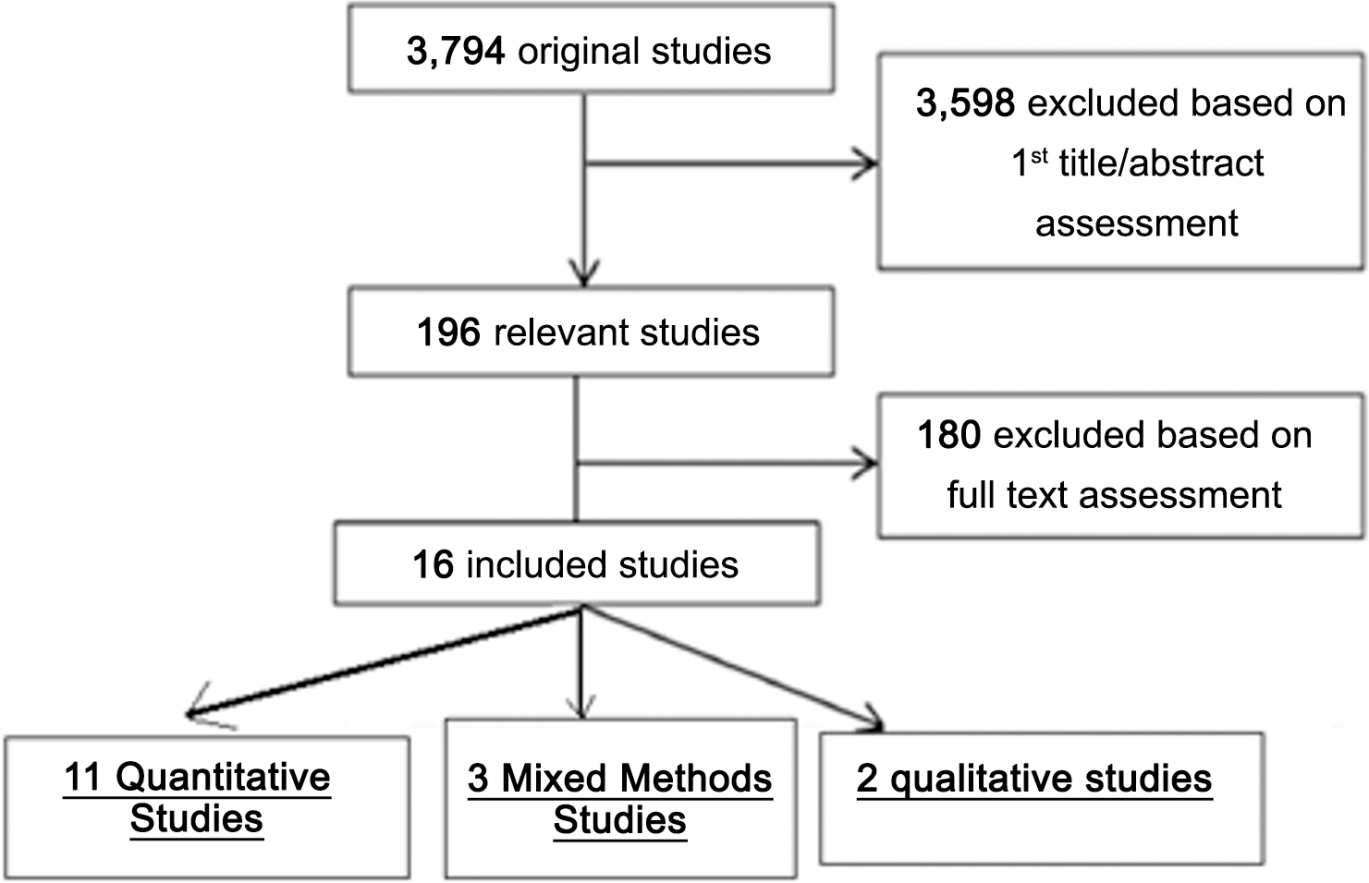
Flow diagram of included studies.

The eligibility criteria for included studies are shown in Table 1 below. Fig 1 explains the procedure for selecting studies included in the review.

**Table 1:** Eligibility Criteria.

***Types of participants***
1. Any person of any age who is already hypertensive or at risk of hypertension
2. Health care providers (any one trained in the biomedical perspective)
3. Local health care providers such as complementary and alternative medicine doctors, traditional healers and patent medicine vendors
***Study outcome/focus***
Beliefs or perceptions of individuals on hypertension assessed either directly (i.e. with no outcome measure) or indirectly (with outcome measure e.g. adherence to medications or use of CAM)
***Types of studies***
1. Qualitative, quantitative observational and mixed methods studies that assesses believes or perceptions of individuals on hypertension either directly (i.e. with no outcome measure) or indirectly (with outcome measure e.g. adherence to medications or use of CAM)
2. Studies were included regardless of study quality
3. No publication date or language barrier was imposed
4. Grey literature such as conference abstracts and unpublished reports were included

## Data Collection and Data Item

Data were extracted independently by 2 reviewers (JA and PF) using a form that was piloted on 3 selected studies. The following items were extracted from each study: characteristics of study [Study design (quantitative or qualitative), setting, characteristics of participants and objectives]; EM categories and beliefs of participants under each EM category assessed in the study, prevalence of these reported beliefs, information on use of theoretical model tools or any other validated tools to assess EM.

## Assessment of Study Quality

Study quality and potential bias was assessed independently by 2 authors (JA, PF) by applying appropriate quality criteria for each type of study (qualitative, quantitative, mixed methods) using validated tools of existing framework. For primary qualitative and quantitative studies, the Critical Appraisal Skills Program (CASP) checklist was used to assess their quality as shown in S6 and S8 Tables respectively [28]. This framework was selected based on its use in previous systematic reviews, simplicity and rapid application of tool and applicability to the various types of primary qualitative and quantitative studies included in the review [28, 29, 30,31,32,33, 34, 35, 36, 37].

For mixed methods studies, there is currently no consensus on the criteria for appraising quality of this methodology [38, 39] but there are some tools in the literature that have been proposed, many of which are still undergoing criteria refinement, content validity and reliability testing [40, 41, 42]. For this study, we used the current 2011 version of Mixed Method Appraisal Tool (MMAT) to assess quality of mixed method studies because its recommendations are based on rigorous literature review and have been revised using feedbacks from workshops for content validation [43]. It has also been used in at least fifty systematic mixed studies reviews worldwide [44].The results of quality assessment for mixed methods studies is presented in S7 Table. S1 Fig shows the original 2011 version of the MMAT.

Any discrepancies were resolved by RM and NF who are specialists in qualitative and quantitative studies respectively.

## Data Synthesis and Analysis

Selected articles were categorised as primary qualitative, quantitative or mixed methods studies according to the authors’ descriptions and were arranged according to the theoretical framework for lay persons and health care providers respectively. All studies were included irrespective of methodological quality.

Qualitative evidence was derived from primary qualitative studies and qualitative components of mixed method studies while quantitative evidence was derived from primary quantitative studies and quantitative component of mixed method studies [40, 45].

Qualitative and quantitative data were integrated in the discussion section assuming a complementary stance i.e. Qualitative and quantitative data are treated separately but each component (qualitative or quantitative) adds to one another [40, 45].

Using a narrative synthesis, qualitative data were used to modify and group data into 6 EM categories (“definition”, “cause”, “course”, “patho-physiology”, “symptoms” and “treatment”) while quantitative data were used to measure the frequency of beliefs under the EM categories. Two reviewers (JA & PF) independently assigned belief items under the EM categories. Disagreements were resolved by a third reviewer (RM).

### Qualitative Analysis

Findings from qualitative studies were analysed using descriptive analysis reporting format in how many beliefs under the EM categories arose with specific examples of belief statements to clarify EM categories.

### Quantitative Analysis

The proportion of individuals who expressed a particular belief under each EM category was extracted from each study. This resulted in a frequency measure of how popular a belief is and facilitated identification of beliefs that might need addressing when seeking behavioural change for particular outcomes like adherence to medication.

## Results

### Study selection

Fig 1 shows a flow chart of included studies. A total of 3,794 articles were identified. Of these, 3,598 papers were excluded based on review of title/abstracts. Then, the full texts of the remaining 196 articles were examined in more detail, of which 16 studies (2 qualitative, 11 quantitative and 3 mixed methods studies) were included in the review.

### Study Characteristics

A Summary of study characteristics is presented in Table 2 while detailed characteristics are presented in S3, S4 and S5 Tables.

**Table 2:** Study characteristics (n = 16).

Number of studies				
	Qualitative	Quantitative	Mixed Methods	Total
**Geo-political zones**				
North- East(NE)	0	0	0	0
North -West(NW)	0	1	0	1
South- West(SW)	1	5	3	9
South- South(SS)	0	0	0	0
South- East(SE)	0	3	0	3
North-Central(NC)	1	2	0	3
**Study Setting**				
Primary health centre	0	1	0	1
Secondary/tertiary health centre	2	5	0	7
Community	0	5	3	8
**Study type**				
Focus groups	0	0	1	1
In depth interview	1	0	1	2
Focus groups & Interviews	1	0	0	1
Cross Sectional	0	11	1	12
**Study population**				
Only hypertensive patients	2	7	1	10
Physicians Only	0	1	0	1
Other health care workers (nurses, CHEW, JCHEWS…..)*	0	0	1	1
Traditional healers (CAM healers, patent medicine vendors….)**	0	0	1	1
Non hypertensive patients (but at risk)	0	3	0	3
**Total**	**2**	**11**	**3**	**16**

*CHEW = community health extension workers**, JCHEW =** junior community health extension worker.

** **CAM healers =** complementary and alternative medicine healers.

## Results of Individual Studies and Synthesis of Results

Another EM (“definition”) was added to the Kleinman’s explanatory model for hypertension. This is because a good number of the studies included in this review explored this concept as a separate entity. In addition, subsuming it under the 5EMs contained in Kleinman’s original model would obscure a great deal of information that might potentially be important in the understanding of explanatory models of Nigerians about hypertension [26, 46, 47, 48, 49].

Across all studies, one aspect of EM was particularly absent: Pathophysiology or biomedical explanation of the disordered activities that occurs in a disease or injury. In contrast, perceptions of cause, course, symptoms and treatment of hypertension were expressed fairly frequently by all categories of participants in most studies. The definition of hypertension or “what hypertension is” was explored in only few studies.

The content analysis of the qualitative data in which textual data is assigned to EM categories is presented in S9 Table

### Health care provider reported beliefs in qualitative studies

Perception of health care providers about hypertension was explored in the qualitative component of one mixed methods study [50]. The study explored EM about hypertension amongst nurses in a primary health care centre.

#### Cause

Health care providers (Nurses) reported that although the cause of hypertension was unknown, stress was a major risk factor for acquiring the disease. In addition, other risk factors such as increased salt intake, heredity, lack of physical activity, poor diet (including increased fat intake, low consumption of fruits and vegetables) were also cited.

#### Course of illness

Health care providers (Nurses) reported that hypertension was a chronic disease and that it requires continuous follow up for life [50].

#### Symptoms and treatment

The perception of symptoms and treatment of hypertension was not explored among health care providers (nurses) in this study.

### Lay health care providers’ reported beliefs in qualitative studies

Lay health care providers’ (CAM practitioners and PMV) perception about hypertension varied by study and within study. Most participants, especially CAM healers had different views on the cause, course, symptoms and treatment of hypertension.

#### Cause

When asked what causes hypertension, some CAM practitioners reported that hypertension was caused by evil spiritual forces while others felt it was caused by poverty and too much blood in the body. Most PMVs said that smoking, high salt intake, low physical activity levels were prominent causes of hypertension [50, 51].

#### Course of illness

All PMVs stated that hypertension was not curable but perception of curability varied among CAM healers. However, both CAM healers and PMVs believed that untreated hypertension could lead to complications such as stroke and heart failure [50, 51].

#### Symptoms

When asked about symptoms of hypertension, no participant said it was symptomless but some, especially PMVs believed that headache and palpitation could be some of the symptoms of hypertension. Other symptoms of hypertension that were expressed by participants include chest pain, general weakness and loss of weight [51, 52].

#### Treatment

Most CAM healers believe that hypertension is curable by adhering strictly to using their herbal remedies and instructions [50, 52]. A few other CAM healers reported that they were not sure if hypertension was curable but they asserted that their remedies will help reduce the negative effects of hypertension [50]. Interestingly, across all studies, traditional healers do not consider themselves as the only care giver for hypertensive patients. Most of them reported that they would sometimes refer their clients to the hospital to check their blood pressure and would refer them to the hospital if their condition became very critical; for example, when they present with complications such as stroke [50, 51].

### Hypertensive patients’ reported beliefs in qualitative studies

Perception about hypertension among hypertensive patients were explored by all primary qualitative studies and qualitative components of the 2 mixed methods.

#### Definition

Across all studies, most patients were able to identify their illness as hypertension, high BP or high blood pressure. However, one study reported variability in the meaning of hypertension and high blood pressure. While some reported that hypertension was a severe form of high blood pressure others said hypertension was the medical term for the lay word: “high blood pressure” [26].

#### Cause

Perception about causes of hypertension varied by study and within study. Some studies asked questions about causes of hypertension separate from risk factors while some did not. But in this review, all responses regarding causes and risk factors are grouped under cause EM category. Many reported that hypertension was caused by stress, thinking too much and lack of social infrastructure [26, 50, 51, 53]. A number of participants asserted that hypertension was caused by the devil or some evil spirit [52].

Regarding risk factors for development of hypertension, some participants believed that high salt intake, smoking, excessive alcohol consumption, low levels of physical activity, high fat diet, and obesity could be potential risk factors but many others did not think so. In one study, a number of participants believed that obesity was a sign of good living and affluence and that weight loss was a sign of disease, poverty or miserliness [53].

#### Course

A number of participants expressed the belief that hypertension is temporary and not chronic. Very few thought that it was a chronic disease. In one study, some also reported that it could lead to stroke and sudden death [53].

One study showed gender specific motivation for taking drugs in that some men reported that they took their drugs regularly because they believe that non-adherence could lead to loss of libido and erectile dysfunction [26].

#### Symptoms

Most participants reported headache, sleeplessness, dizziness, weakness and palpitations as the commonest symptom of hypertension [26, 53].Some also cited pain in the legs, chest and arms as symptoms. Very few asserted that it could be asymptomatic.

#### Treatment

Regarding behaviours, activities, agents or recommended actions that are believed to be effective in the treatment of hypertension, participant’s view and activities varied across and within studies. Beliefs about which treatment was effective was mostly linked to their perception of the cause of hypertension and affected their adherence to such treatment modality. Moreover, other factors also determined adherence to treatment.

Some of the participants believed that orthodox drugs alone were effective in managing hypertension but could not cure it.Others reported that taking orthodox medicine and CAM simultaneously is more effective in curing hypertension [26, 50, 51, 53].

Generally, across all studies, most participants believe that it can be cured whereas a few assert that it could not be cured [26, 50, 51, 53].

Some believed that only prayer can cure hypertension because it is caused by an evil spirit, as such only God can heal the disease [50].

Regarding choice of medication, all participants deferred that decision to their physicians. Most believed that their doctors knew what was best for them.

A few participants across all studies believed and practiced behaviours such as not adding salt to food, not using tobacco, irregular alcohol consumption, weight reduction programmes, regular exercises, regular check of blood pressures and consumption of fruits and vegetables [26, 50, 51, 53].

### Health care provider reported beliefs in quantitative studies

Health care providers reported beliefs on hypertension was explored in only 1 quantitative study and was conducted on physicians (medical doctors) only.

#### Definition

There were wide differences in beliefs about the cut off for hypertension in the general population. Just over half (55%) of the physicians believed that 140/90 mmHg was the correct benchmark for non complicated hypertension. Some believed that 130/80mmHg was the benchmark while a few (1%) believed that 120/75mmHg was a more appropriate benchmark [54].

#### Cause

Half of the physicians believe that stress was a contributory factor in the development of hypertension among other factors [54].

#### Treatment

A large number of physicians (76.2%) do not believe in the use of herbal preparations in the treatment of hypertension. But, about 13% of physicians still prescribe herbal drugs alongside orthodox drugs for their patients [54].

### Lay health care providers’ reported beliefs in quantitative studies

There was no quantitative study conducted on lay health care providers (CAM practitioners and PMVs).

### Lay Persons’ reported beliefs in quantitative studies

Table 3 presents a summary of the proportions of participants with their beliefs about hypertension.

**Table 3:** Proportion of Lay persons with respective beliefs under EM categories: “definition”, “cause”, “Course”, “symptoms” &” treatment” in quantitative studies.

EM Categories	Females% (Reference)	Males % (Reference)	Females &Males %(Reference)
**DEFINITION**			
- Raised Blood Pressure	**112/252 (44.4%) [47]**		**445/1365(32.6%)[46]; 117/254(46.1%)[57]; 164/252(65.1%) [49]; 28/2000(1.4%) [48]**
- Psycho-social stress			**366/1365(27%) [46]**
- Others			**587/1365(43%) [46]**
**CAUSES**			
-Psycho social stress(reported as risk)	**178/252(70.6%) [47]**		**854/2000(42.7%)[48]; 183/275(66.5%)[56]; 1229/1365(90%)[46]**
-obesity	**167/252(66.3%) [47]; 30/101(30.1%) [55]**	**40/101(40%) [55]**	**887/1365(65%)[46]; 66/101(65%) [55]; 32/2000(1.6%)[48]; 169/275(61.5%)[56]**
-smoking	**137/252(54.4%) [47]; 25/101(25%) [55]; 73/146(50%) [59]**	**30/101(30.1%) [55]; 70/114(61.4%)[54]**	**875/1365(64%)[46]; 54/101(53%)[55]; 725/2000(36.2%)[48]; 185/275(67.5%)[56]**
-reduced physical activity	**7/101(7%) [55]**	**9/101(9%)[55]**	**11/101(11%) [55]; 24/2000(1.2%)[48]; 31/252(12.3%) [49]**
-excessive alcohol intake	**120/252(47.6%) [47]; 16/101(16%) [55]; 85/146(58.2%)[59]**	**20/101(20%) [55]; 84/114(73.7%)[59]**	**4/101(4%) [55]; 183/275(66.5%)[56]; 99/252(39.3%) [49]; 975/1365(71%)[46]**
-excessive salt intake	**40/101(40%) [55]; 195/252(77.4%) [47]**	**50/101(50%) [55]**	**1004/1365(76%) [46]; 85/101(85%) [55]; 220/252(87.3%) [49];56/2000(2.8%)[48]**
-high fat diet	**195/252(77.4%) [47]; 89/146(61%)[59]**	**67/114(58.8%)[59]**	**894/1365(65%)[46]; 22/2000(1.1%) [48]; 42/252(16.7%)[49]**
-family history	**185/252(73.4%) [47]**		**98/1365(7.2%)[46]; 30/260(11.5%)[59]**
-Race (being black)			**120/254(47.2%) [57]**
-low fruit and vegetable intake	**18/101(18%)[55]**	**15/101(15%) [55]**	**25/101(25%) [55]; 35/2000(1.7%) [48]; 60/252(23.8%) [49]**
-Psychosocial stress(reported as cause)			**614/1365(45%)[46]; 130/260(50%)[59]**
-Evil curses/spirits/charms	**67/252(26.6%) [47]**		**68/1365(5%)[46]; 5/260(1.9%)[59]**
**SYMPTOMS**			
-Asymptomatic	**155/252(61.5%) [47]**		**47/275(17.1%)[56]**
-headache	**59/252(23.4%) [47]**		**213/275(77.5%)[56]**
-internal heat	**38/252(15.1%) [47]**		
Dizziness			**153/275(55.6%)[56]**
Chest pain			**155/275(56.4%)[56]**
Palpitations			**114/275(41.5%)[56]**
**COURSE**			
**Question/Item assessed in studies**			
Do you believe not taking your medications poses an increased risk for developing complications?			**Said YES– 21.63/30(76.1%) [58]; 59/252(23.4%) [49]**
What are your fears about hypertension?			
Heavy financial burden			**137/1365(10%) [46]**
Heart attack			**68/1365(5%) [46]; 135/254(53.2%)[57]**
Stroke			**546/1365(40%)[46]; 135/254(53.2%)[57] 231/275(84%)[56]**
Kidney failure			**205/1365(15%)[46]; 135/254(53.2%)[57]; 15/275(5.5%)[56]**
Sudden death			**341/1365(25%)[46]; 191/275(69.5%)[56]**
**TREATMENT**			
**Participants who thought hypertension could be cured**			**887/1365(65%)[46]; 164/254(64.6%)[57]; 162/252(64.1%) [55]**
**Participants methods for control of hypertension**			
Homeopathic medicine			**411/1365(30.1%)[46]**
Native doctors/Herbal concoction			**314/1365(23%)[46]**
Prayers and faith healing			**628/1365(46%) [46]**
Orthodox drugs	**190/252(75.4%) [47]; 7/59(11.8%)[48]**	**4/59(6.8%)[48]**	**11/59(18.6%) [48]**
**Activities participants engage in for prevention or control of hypertension**			
Non-addition of extra salt	**124/252(49.2%) [47]; 17/59(28.8%)[48]**	**13/59(22%) [48]**	**65/101(65%) [49]; 144/252(57.1%)[55]; 30/59(50.8%)[48]**
Weight reduction	**108/252(42.8%) [47]; 5/59(8.4%)[48]**	**2/59(3.3%)[48]**	**9/101(9%) [49]; 108/252(42.9%)[55]; 7/59(11.9%)[48]**
Non-tobacco use/smoking	**213/252(84.5%) [47]; 12/146(8.2%)[54]**	**26/114(22.8%)[59]**	**8/101(8%) [49]**
Reduced consumption of alcohol	**213/252(84.5%)[47]; 32/146(21.9%)[54]**	**61/114(53.5%)[59]**	**32/101(32%) [49]; 74/252(29.4%)[55]**
Consumption of vegetables/fruits	**174/252(69%) [47]**		**25/101(25%) [49]; 33/252(13.1%)[55]**
Regular exercise	**144/252(57.1%) [47]; 4/59(6.7%)[48]; 61/146(41.8%)[54]**	**3/59(5%)[48];45/114(39.5%)[59]**	**9/101(9%) [49]; 16/252(6.3%)[55] 7/59(11.9%)[48]**
Regular BP check	**108/252(42.9%) [47]; 2/59(3.3%) [48]**	**2/59(3.3%)[48]**	**4/59(6.7%) [48]**
Reduced consumption of fat in diet	**2/59(3.3%)[48]; 83/146(56.8%)[59]**	**62/114(54.4%)[59]**	**2/59(3.3%) [48]; 22/252(8.7%) [49]**
Reduced stress	**18/59(30.5%)[48]**	**20/59(33.8%)[48]**	**38/59(64.4%)[48]**

### Hypertensive and non-hypertensive individuals

Among the 10 studies that explored EM of lay persons, 7 of them strictly included hypertensive patients while 3 studies included both hypertensive and non-hypertensive individuals. But, no attempt was made by the studies to differentiate responses of hypertensive from non-hypertensive individuals. So findings are presented generally regardless of their blood pressure status i.e. hypertensive or not

#### Definition

Across all studies that asked what hypertension meant, the proportion of participants’ who said that hypertension meant raised blood pressure ranged from 1.4% [48] to 65.1% [49]. In the study that was conducted on females only, 44.4% of them believed that hypertension meant raised blood pressure [47]. In the study conducted by Oke et al. (2004), 27% of participants thought that hypertension simply meant psychological stress. Other Beliefs elicited include: hypertension is a heart disease (4%), hypertension is nervousness (4%) and hypertension is palpitations (4%) [46].

#### Cause

Regarding beliefs about causes of hypertension, it is important to note that, in most quantitative studies the question about risk factors was asked separately from causes and some responses such as psychological stress overlap. But responses regarding risk factors and causes were reported together under the “cause” EM category.

For example, a large number of participants ranging from 42.7% [48] to 90% [46] believed that psychological stress was a major risk factor for hypertension. But, only about half of participants in two studies believed that stress was a cause of hypertension [46]. A few of the participants in three studies, 1.9%, 5% and 26.6% respectively believed that evil spirits, charms or curses were the cause of hypertension [46, 47].

#### Symptoms

Two studies assessed participants’ belief about symptoms of hypertension. The majority of participants said that headache was the main presentation of hypertension [47, 55, 56]. But in one study a considerable number (61.5%) thought that hypertension is asymptomatic [47].

#### Course

In most studies, beliefs about course of hypertension were extracted from motivations for taking prescribed orthodox drugs. In other words, perception of what will happen if hypertension was left to go untreated.

Motivation for taking prescribed orthodox drugs varied among participants in different studies. The most common motivation was the fear of developing stroke. This was expressed by an average of 60% of participants across 3 studies [46, 55, 56,]

#### Treatment

In this review, it is important to note that it was assumed that; what people used in the treatment of hypertension i.e. their practices could be expressed as a measure of their EM or perception of treatment of hypertension.

Across 3 different studies, approximately 65% of participants believe that hypertension could be cured [57, 58, 59].

In one study, as much as 46% use faith healing and prayers alone as a means of control of hypertension [46]. Notably, there was generally a low rate of use of orthodox medicine alone in the treatment of hypertension. Conversely, in the study conducted by Azubike et al. (2014) [47] on women, 75.4% used orthodox drugs only compared to 11.8% in another study by Oladapo et al. (2013) [48].

Across all studies, regarding activities for the prevention or control of hypertension, there were generally low numbers of participants who practiced activities such as regular BP check, regular physical exercise, reduced fat intake, reduced consumption of alcohol and increased consumption of fruits and vegetables.

Relatively similar proportions of women and men practiced regular BP check and reduced consumption of fat diet [48, 55,59]. But over 40% of women practiced regular BP check in the study conducted among female [47].

### Comparison of health beliefs among ethnic groups

Although a few studies [26, 50, 51, 52, 53,59] collected data on ethnic groups of the participants, none of the studies reported beliefs about hypertension according to ethnic group.

## Discussion

### Summary of evidence

Among primary qualitative and qualitative component of mixed method studies, explanatory models of healthcare providers on hypertension were scarcely explored. This was also the case among quantitative studies where only 1 study assessed the beliefs of physicians about hypertension. Although, some studies on explanatory model of HCP on hypertension have been done in some other parts of the world, there appears to be a general paucity of these studies globally and in Nigeria [17, 18, 19, 20]. This is probably because it is assumed that since HCPs have been trained in the biomedical perspective, their perception should align entirely with the biomedical model. But, this may not always be the case. For example, according to the biomedical model, evidence based data show that in 95% of cases, the cause of hypertension is unknown but that there are several risk factors which increase the risk of development of hypertension. Although it has been shown that links exist between increased risk of coronary heart disease and stress, there is no strong evidence yet that stress causes hypertension [60]. But, our review showed that in the quantitative study, most HCPs (medical doctors) believe that stress is a major cause of hypertension. The qualitative study conducted on nurses did not explore their perception on causes of hypertension so it difficult to make comparison between nurses and other HCPs. Furthermore, a small fraction believe that herbal medications could help in the control of hypertension and actually prescribe it for their patients but majority of them do not believe in or prescribe herbal medications. In the future qualitative studies should explore the reason for the explanatory model of stress as cause of hypertension among HCPs.

Perhaps the most surprising observation on perception of HCPs in Nigeria on hypertension is that their response to the question on blood pressure (BP) cut-off point for primary uncomplicated hypertension differed widely. Because no qualitative study explored the definition of hypertension among HCPs, it is difficult to ascertain whether this was a lack of knowledge problem or a contextual explanatory model of the physicians involved. Moreover, these findings are similar to reports from 2 different studies in Iran and Saudi Arabia respectively where knowledge about BP cut-offs differed among physicians [61, 62]. Although, this may be attributed to multiple guidelines or personal experiences of HCPs, these findings are from a study with a low power, hence it is difficult to generalise. However, these differences in perspective highlight an important subject of concern and require further probing so as to have unified monitoring indicators for the management of hypertension in Nigeria.

For traditional healers (CAM healers and PMVs), their beliefs were explored only through qualitative studies. PMVs form an important part of the informal healthcare sector in Nigeria. PMVs beliefs about hypertension seem to be relatively similar to health care professionals in areas of risk factors for hypertension, course of hypertension (i.e. chronic nature of disease) and methods of treatment. This may be partly because most PMV sell orthodox drugs and are inclined to recommend it to their clients so as to make profit. However, what they actually believe and practice for themselves may not be different from other persons on the street.

Explanatory models of hypertension differed widely between CAM healers. Although, some of them believed that hypertension could be caused by stress- a statement that resonated across all categories of participants; most differed in their beliefs about other risk factors for hypertension and treatment modalities. The reason for wide variations in beliefs about hypertension among CAM healers in the same geo-political zone of the country is unknown. This may simply be due to multiple ethnicities within geo-political zones but there may be others factors responsible for this and therefore requires further probing.

For hypertensive and non-hypertensive patients, all study types including quantitative, qualitative and mixed methods studies explored beliefs about hypertension. Even though, most qualitative studies were not conducted in the same community or similar populations as the quantitative studies, the wide variety of studies gave opportunity for contrasting findings of quantitative studies with views of participants from qualitative studies. In our review, most of the findings of qualitative studies were similar to those obtained from quantitative studies. However, the proportion of people with similar explanatory models differed widely across different quantitative studies. This could be as a result of the wide variations in the study design and methodologies of the quantitative studies and heterogeneity of study populations, especially in the way questions were asked. However, with these inadequacies it was still possible to group findings from studies under the respective explanatory model categories and make inferences on which belief item was most popular.

Beliefs of Nigerian hypertensive and non- hypertensive patients about hypertension were relatively similar to those reported in a study on African-American patients [19]- particularly in that misconceptions about the meaning of hypertension was quite high.

Across all studies included in the review, some Nigerians believed that life style habits such as alcohol intake, exercise levels, cigarette smoking were risk factors for developing hypertension.But in studies that compared beliefs about lifestyle habits versus practice of such habits, there was always a low rate of practice of life style modification compared to beliefs in the quantitative studies. The reasons for this discordance may be due to several factors that may be explained by the Necessity Concerns Model (NCF).Future studies applying the NCF model to different groups of patients may reveal specific factors that if properly addressed may help improve adherence.

However, overall, there appear to be more women practicing life style modifications such as non-tobacco use/smoking, reduced consumption of alcohol, consumption of vegetables/fruits, regular exercise, regular BP check, reduced consumption of fat in diet compared to men. Understanding these differences may help develop interventions that are appropriate for management of hypertension in Nigeria.

Although very few studies have been conducted on beliefs of Nigerians about hypertension, our review brings together what is available and identifies gaps for future research. As postulated by the knowledge translation model, success is probable if interventions are tailored to evaluations of barriers and facilitators [63]. This review aids this process by pooling together beliefs of individuals which is an important individual level barrier to control of hypertension.

### Study strengths and Limitations

To our knowledge, no systematic review of the literature on beliefs of hypertension has been published to date. The current review reveals both similarities and differences in the beliefs between laypersons and health care providers and identifies misperceptions that should be addressed when designing interventions for hypertension control in Nigeria.

Moreover, this review highlights an important gap in the literature on perceptions of different ethnic groups on hypertension. Our review showed that most studies were conducted in urban areas such as state capitals that have populations that are ethnically diverse. Very few studies were conducted in rural areas that had homogenous ethnic group participants. This limited our ability to generalise findings because of potential differences influenced by ethnicity. This underscores the need for future research in rural communities and in rural health care facilities such as primary health care centres to identify specific explanatory models that might help in planning appropriate programs.

A unique strength this review presents is the systematic analysis of qualitative and quantitative studies. This enabled simultaneous comparison of findings. Although findings from quantitative studies were from heterogeneous study designs, they were mostly corroborated by results of entirely different qualitative studies. However, the qualitative studies were not always conducted among similar populations as the quantitative studies thereby making generalizability difficult.

One important limitation of this review is the assumption made about quantitative studies.

First, extracting the responses of knowledge of definitions, causes and courses from KAP surveys to inform explanatory models or perceptions about hypertension may not give accurate culture specific notions of the illness, especially because the intention of the original researcher might have been different. For example, some studies have pointed out that public health professionals usually share the view that knowledge and beliefs are different. Knowledge to them is usually based on universal truths (biomedical ideas) while beliefs are erroneous ideas that are different from biomedical idea, and as such are barriers to achieving health promoting behaviours [64, 65]. But anthropologists do not consider knowledge different from belief. This is because the role of knowledge or beliefs the community possesses cannot be overemphasized in achieving control of diseases [65]. Although, sometimes, it may be difficult to elicit culture specific beliefs about illnesses in a KAP survey format, this review reveals information for further exploration.

Second, in this review knowledge of risk factors were grouped together as causes for ease of analysis. However, most of the quantitative studies separated the risk factors questions from cause questions with some items overlapping both domains. For example the word psychological stress appeared as an option for risk factor and cause of hypertension in different studies. One problem with this arrangement is that it is likely that in answering the questionnaires, the differences between what constitute a risk factor or a cause was not well explained to participants. Therefore distinct demarcation between these concepts could not be made.

Furthermore, the response to knowledge questions on definition, cause, course and symptoms framed in an ordinal scale format may not be representative of the true situation. But relative agreement between findings from quantitative and qualitative studies included in this review may give some confidence about reported beliefs.

In the same vein, the assumption that what people used in the treatment of hypertension i.e. their practices could be expressed as a measure of their EM or perception of treatment of hypertension was quite audacious. This is because people may use a particular method of treatment for other reasons than their belief in it. But the results from qualitative studies about treatment beliefs were mostly congruent with the findings from quantitative studies.

The quality of the methodology employed in the study designs of qualitative, quantitative and mixed method was moderate. Not all studies used validated instrument for collecting data. In addition, most studies assessed beliefs using different question types and measurements. Further, some studies focused strictly on barriers to outcomes such as adherence to medications. Hence not all aspects of the aforementioned explanatory model categories were explored in many studies.

Another potential limiting factor is the small number of studies in the review and the contrasting age of participants across studies included in the review. Most studies did not investigate associations between age and the knowledge and awareness, attitudes and practices. Therefore a Meta analysis of the quantitative findings was not conducted.

It was also not possible to identify representativeness of studies included in the review as it was not always mentioned which socio-economic environment the studies were conducted (for example rural or urban). This factor may affect beliefs but this was not assessed in most studies.

## Conclusion/Implications

In order to achieve control of hypertension in Nigeria, interventions should be informed by adequate knowledge of beliefs regarding hypertension across the numerous ethnic groups in Nigeria, settings (rural or urban), age and sex. More methodologically sound studies should be conducted that explores beliefs in these contexts so as to improve our basis for determining the most important belief factors for control of hypertension and develop interventions appropriate for different settings in Nigeria.

## Supporting Information

S1 PRISMA Checklist (DOC)

S1 Table Search strategy for identification of all types of studies (Medline). (DOC)

S2 Table Sensitive search strategy specifically for qualitative studies (Medline). (DOC)

S3 Table Detailed study characteristics of qualitative studies. (DOC)

S4 Table Detailed study characteristics of Mixed Methods Studies. (DOC)

S5 Table Detailed study characteristics of quantitative Studies. (DOC)

S6 Table Quality appraisal of qualitative studies. (DOC)

S7 Table Quality appraisal of mixed methods studies using MMAT (adapted from MMAT-Version 2011) see original tool from MMAT website below. (DOC)

S1 Fig Complete MMAT- VERSION 2011. (DOC)

S8 Table Quality appraisal of quantitative studies. (DOC)

S9 Table Examples of beliefs statements on hypertension per EM among qualitative studies. (DOC)
