# Supplementary material for: Beliefs of Health Care Providers, Lay Health Care Providers and Lay Persons in Nigeria Regarding Hypertension. A Systematic Mixed Studies Review
Source: PLoS One. 2016 May 5;11(5):e0154287. doi: 10.1371/journal.pone.0154287 (PMC4858295; doi:10.1371/journal.pone.0154287)
Supplement: S1 Table — (DOC) [file pone.0154287.s002.doc]

**S1 Table: search strategy for identification of all types of studies (Medline)**

|  | **Searches** | **Results** |
| --- | --- | --- |
| 1 | exp Perception/ | 350145 |
| 2 | beliefs.mp. or exp Culture/ | 150141 |
| 3 | exp Attitude to Health/ or exp Health Knowledge, Attitudes, Practice/ or "lay health belief".mp. | 315630 |
| 4 | "Attitude of Health Personnel"/ or exp Attitude to Health/ or exp Attitude/ or attitude.mp. | 460017 |
| 5 | exp Adult/ or exp Adolescent/ or meaning.mp. | 6405511 |
| 6 | exp Aged/ or exp Health Behavior/ or exp Models, Psychological/ or exp Health Knowledge, Attitudes, Practice/ or exp Patient Compliance/ or exp Middle Aged/ or exp Adult/ or exp Attitude to Health/ or "health belief".mp. | 6059398 |
| 7 | exp Health Status/ or exp Adult/ or exp Attitude to Health/ or exp Health Behavior/ or exp Adolescent/ or "health behaviour".mp. or exp Middle Aged/ | 6555847 |
| 8 | (health adj1 belief$).mp. [mp=title, abstract, original title, name of substance word, subject heading word, keyword heading word, protocol supplementary concept word, rare disease supplementary concept word, unique identifier] | 3727 |
| 9 | Nigeria$.mp. or exp Nigeria/ | 29307 |
| 10 | exp Aged/ or exp Blood Pressure/ or exp Hypertension/ or exp Middle Aged/ or hyperten$.mp. | 4467617 |
| 11 | "high blood pressure".mp. or exp Hypertension/ | 224241 |
| 12 | exp Adolescent/ or exp Middle Aged/ or exp Diabetes Mellitus, Type 2/ or exp Adult/ or exp Metabolic Syndrome X/ or exp Antihypertensive Agents/ or exp Blood Pressure/ or exp Hypertension/ or "elevated blood pressure".mp. or exp Cardiovascular Diseases/ | 7566136 |
| 13 | exp Aged/ or exp Adult/ or exp Coronary Disease/ or exp Obesity/ or exp Middle Aged/ or exp Antihypertensive Agents/ or exp Blood Pressure/ or exp Hypertension/ or "raised blood pressure".mp. or exp Cardiovascular Diseases/ | 7144915 |
| 14 | 1 or 2 or 3 or 4 or 5 or 6 or 7 or 8 | 6908258 |
| 15 | |  | 10 or 11 or 12 or 13 | | --- | --- | | 7684406 |
| 16 | 9 and 14 and 15 | 14102 |
| 17 | limit 16 to humans | 14007 |
| 18 | limit 17 to (full text and humans) | 1773 |
| 19 | remove duplicates from 18 | 1750 |
