# Supplementary material for: Beliefs of Health Care Providers, Lay Health Care Providers and Lay Persons in Nigeria Regarding Hypertension. A Systematic Mixed Studies Review
Source: PLoS One. 2016 May 5;11(5):e0154287. doi: 10.1371/journal.pone.0154287 (PMC4858295; doi:10.1371/journal.pone.0154287)
Supplement: S2 Table — (DOC) [file pone.0154287.s003.doc]

**S2 Table: sensitive search strategy specifically for qualitative studies (Medline)**

|  | **Searches** | **Results** |
| --- | --- | --- |
| 1 | Qualitative Research/ | 23815 |
| 2 | Nursing Methodology Research/ | 15759 |
| 3 | Questionnaires/ | 326469 |
| 4 | exp Attitude/ | 281076 |
| 5 | Focus Groups/ | 18584 |
| 6 | discourse analysis.mp. | 1053 |
| 7 | content analysis.mp. | 13897 |
| 8 | ethnographic research.mp. | 582 |
| 9 | ethnological research.mp. | 7 |
| 10 | ethnonursing research.mp. | 29 |
| 11 | constant comparative method.mp. | 1010 |
| 12 | qualitative validity.mp. | 11 |
| 13 | purposive sample.mp. | 2007 |
| 14 | observational method$.mp. | 491 |
| 15 | field stud$.mp. | 10954 |
| 16 | theoretical sampl$.mp. | 398 |
| 17 | phenomenology/ | 0 |
| 18 | phenomenological research.mp. | 297 |
| 19 | life experience$.mp. | 3403 |
| 20 | cluster sampl$.mp. | 4563 |
| 21 | or/1-20 | 590727 |
| 22 | hypertension/ | 200888 |
| 23 | Nigeria$.mp. or exp Nigeria/ | 29307 |
| 24 | 21 and 22 and 23 | 59 |
| 25 | Remove duplicates from 24 | 59 |
