# Supplementary material for: Beliefs of Health Care Providers, Lay Health Care Providers and Lay Persons in Nigeria Regarding Hypertension. A Systematic Mixed Studies Review
Source: PLoS One. 2016 May 5;11(5):e0154287. doi: 10.1371/journal.pone.0154287 (PMC4858295; doi:10.1371/journal.pone.0154287)
Supplement: S3 Table — (DOC) [file pone.0154287.s004.doc]

**S3 Table**: detailed study characteristics of qualitative studies

| **Study, Year, Participants** | **Region** | **Ethnic groups** | **Recruitment site** | **Study Focus** | **Population** |
| --- | --- | --- | --- | --- | --- |
| **Taylor et al,2012,Lay persons (patients)** | South-West | Yoruba, Ibo, others | Lagos University Teaching Hospital | To elicit patients' beliefs regarding the meaning, causes, symptoms and treatment of HTN using explanatory model | Diagnosed and treated for HTN |
| **Odusola et al, 2014, Lay persons (patients)** | North- Central | Yoruba, Nupe, Others | Ogo-Oluwa Hospital | to explore patients’ perceptions on inhibitors and facilitators for adhering to hypertension treatment | Diagnosed and treated for HTN |
