# Supplementary material for: Beliefs of Health Care Providers, Lay Health Care Providers and Lay Persons in Nigeria Regarding Hypertension. A Systematic Mixed Studies Review
Source: PLoS One. 2016 May 5;11(5):e0154287. doi: 10.1371/journal.pone.0154287 (PMC4858295; doi:10.1371/journal.pone.0154287)
Supplement: S4 Table — (DOC) [file pone.0154287.s005.doc]

**S4 Table**: detailed study characteristics of Mixed Methods Studies

| **Study, Year, Participants** | **Region** | **Ethnic groups** | **Recruitment site** | **Study Focus** | **Population** |
| --- | --- | --- | --- | --- | --- |
| **Osamor et al, 2010 , Lay persons (patients & CAM practitioners),** | South-west | Yoruba, Ibo, Isoko | Idikan Community | -To investigate the frequency and factors associated with use of CAM among hypertensive subjects in an urban Nigerian community  -To obtain Perspectives about the management of hypertension from CAM practitioners in the community. | - CAM practitioners - Diagnosed and treated for HTN |
| **Osamor et al, 2011, Lay persons(patients)** | South-west | Yoruba, Ibo, Isoko | Idikan Community | To describe treatment-compliance patterns among hypertensive subjects in a Nigerian community and investigates the factors associated with good compliance, including demographic factors, beliefs about hypertension, and the availability of social support | Diagnosed and treated for HTN |
| **Osamor, 2011,**  **- Lay persons (patients, traditional healers & PMV)**  **- Health Care Practitioners (HCP)** | South-west | Yoruba, Ibo, Isoko | Idikan Community | To study care seeking behavior for hypertension in an urban Nigerian community. | -Diagnosed and treated for HTN  - Patent Medicine Vendors(PMV)  -Health Care Practitioners |
