# Supplementary material for: Beliefs of Health Care Providers, Lay Health Care Providers and Lay Persons in Nigeria Regarding Hypertension. A Systematic Mixed Studies Review
Source: PLoS One. 2016 May 5;11(5):e0154287. doi: 10.1371/journal.pone.0154287 (PMC4858295; doi:10.1371/journal.pone.0154287)
Supplement: S5 Table — (DOC) [file pone.0154287.s006.doc]

**S5 Table**: detailed study characteristics of quantitative Studies

| **Study, Year, Participants** | **Region** | **Ethnic Groups** | **Recruitment site** | **Study focus** | **population** |
| --- | --- | --- | --- | --- | --- |
| **Atulomah et al 2010, Lay persons(patients)** | South-west | NA | Univ. Teaching hospital, Ogun | to collect information about perceived severity and threat to life from poor treatment response and medication adherence in hypertensive patients | Diagnosed and treated for HTN |
| **Oke et al 2004, Lay persons (patients)** | South-west | NA | Medical out -patient clinics in 5 hospitals | study of the misconceptions of hypertension by hypertensive patients | Diagnosed and treated for HTN |
| **Azubike et al, 2014,Lay persons(patients)** | North west | Nandu,nimzon, Fulani,hausa, Jaba, others | Primary health care center | To determine the level of knowledge of hypertension and its associated factors, attitudes, practices, and prevalence among rural Nigerian women | Women attending post-natal clinic |
| **Familoni et al 2004,lay persons(patients)** | South-west | NA | Medical out -patient clinics in Univ. teach. Hosp. Ogun | to assess the knowledge and level of awareness of the disease among hypertensive patients | Diagnosed and treated for HTN |
| **Okwuonu et al 2014(a),Lay persons (patients)** | South-east | NA | Olokoro Community | To assess the level of awareness and practice of lifestyle modification among hypertensive adults in a semi-urban community | Diagnosed and treated for HTN |
| **Okwuonu et al 2014(b),Lay persons (patients)** | South-east | NA | Olokoro Community | To identify patient-related barriers to control of hypertension among adults with hypertension in a semi-urban community | Diagnosed and treated for HTN |
| **Ike et al 2010, Lay persons(patients)** | South-east | NA | Cardiac clinics in Univ. teach. Hosp. Enugu | To evaluate the perception, knowledge and practices of Nigerian hypertensive patients regarding hypertension and lifestyle modification measures | Diagnosed and treated for HTN |
| **Oladapo et al 2013, lay persons(patients)** | South-west | NA | Egbeda Local Government area | To assess the level of awareness, basic knowledge and management of hypertension and CV risk factors among people living in rural south western Nigeria | Community Dwellers |
| **Salaudeen et al 2014,lay persons(patients)** | South-west | NA | Ilorin town | To assess the level of knowledge of risk factors among respondents and to compare the blood pressure pattern of bankers and traffic wardens | Bankers and traffic wardens |
| **Adamu et al 2014, HCP** | North-central | NA | Continuing medical education program ,Bida | To assess the knowledge, attitude and practices  of medical offi cers on systemic hypertension in the North-Central region of Nigeria | Medical officers(HCP) |
| **Adeniyi et al 2015, lay persons(patients)** | North Central | NA | University teach. hospital, Jos | To assess hypertension related knowledge levels among hypertensive patients attending tertiary healthcare facilities | Diagnosed and treated for HTN |
