# Supplementary material for: Beliefs of Health Care Providers, Lay Health Care Providers and Lay Persons in Nigeria Regarding Hypertension. A Systematic Mixed Studies Review
Source: PLoS One. 2016 May 5;11(5):e0154287. doi: 10.1371/journal.pone.0154287 (PMC4858295; doi:10.1371/journal.pone.0154287)
Supplement: S6 Table — (DOC) [file pone.0154287.s007.doc]

**S6 Table**: quality appraisal of qualitative studies

| **Study, Year** | **Clear statement/aim of research described** | **Qualitative methodology appropriate** | **Research design appropriate to address aims of research** | **Recruitment strategy appropriate to aims of research** | **Data collected in a way that addressed research issue** | **Reflexivity of the account(i.e. recognition of researcher bias)** | **Ethical issues taken into consideration** | **Data analysis sufficiently rigorous** | **Clear statement of findings** | **How valuable is the research** |
| --- | --- | --- | --- | --- | --- | --- | --- | --- | --- | --- |
| **Taylor et al 2012** | Yes | yes | yes | yes | Yes | No | yes | Yes | yes | yes |
| **Odusola et al 2014** | Yes | yes | yes | yes | Yes | Yes | yes | Yes | yes | yes |
