# Supplementary material for: Beliefs of Health Care Providers, Lay Health Care Providers and Lay Persons in Nigeria Regarding Hypertension. A Systematic Mixed Studies Review
Source: PLoS One. 2016 May 5;11(5):e0154287. doi: 10.1371/journal.pone.0154287 (PMC4858295; doi:10.1371/journal.pone.0154287)
Supplement: S7 Table — (DOC) [file pone.0154287.s008.doc]

**S7 Table**: quality appraisal of mixed methods studies using MMAT (adapted from MMAT-Version 2011) see original tool from MMAT website below

| **Methodological quality criteria** | **Osamor et al, 2010** | **Osamor et al, 2011** | **Osamor, 2011** |
| --- | --- | --- | --- |
| **SCREENING QUESTIONS**  1) Are there clear qualitative and quantitative research questions (or objectives*), or a clear mixed methods question (or objective*)? | Yes | yes | Yes |
| 2) Do the collected data allow /address the research question (objective)? E.g., consider whether the follow-up period is long enough for the outcome to occur (for longitudinal studies or study components). | Yes | yes | Yes |
| **QUALITATIVE**  1)Are the sources of qualitative data (archives, documents, informants, observations) relevant to address the research question(objective)? | Yes | yes | Yes |
| 2) Is the process for analyzing qualitative data relevant to address the research question (objective)? | Yes | yes | Yes |
| 3) Is appropriate consideration given to how findings relate to the context, e.g., the setting, in which the data were collected? | Yes | yes | Yes |
| 4) Is appropriate consideration given to how findings relate to researchers’ influence, e.g., through their interactions with participants? | No | no | No |
| **QUANTITATIVE DESCRIPTIVE**  1) Is the sampling strategy relevant to address the quantitative research question (quantitative aspect of the mixed methods question)? | Yes | yes | Yes |
| 2) Is the sample representative of the population understudy? | No | yes | No |
| 3) Are measurements appropriate (clear origin, or validity known, or standard instrument)? | Yes | yes | Yes |
| 4) Is there an acceptable response rate (60% or above)? | Yes | yes | Yes |
| **MIXED METHODS**  1) Is the mixed methods research design relevant to address the qualitative and quantitative research questions (or objectives), or the  Qualitative and quantitative aspects of the mixed methods question (or objective)? | Yes | yes | Yes |
| 2) Is the integration of qualitative and quantitative data (or results*) relevant to address the research question (objective)? | Yes | yes | Yes |
| 3) Is appropriate consideration given to the limitations associated with this integration, e.g., the divergence of qualitative and quantitative  data (or results*) in a triangulation design? | Yes | yes | Yes |

***** These two items are not considered as double-barrelled items since in mixed methods research, (1) there may be research questions (quantitative research) or research objectives (qualitative research), and (2) data may be integrated, and/or qualitative findings and quantitative results can be integrated
