# Supplementary material for: Beliefs of Health Care Providers, Lay Health Care Providers and Lay Persons in Nigeria Regarding Hypertension. A Systematic Mixed Studies Review
Source: PLoS One. 2016 May 5;11(5):e0154287. doi: 10.1371/journal.pone.0154287 (PMC4858295; doi:10.1371/journal.pone.0154287)
Supplement: S1 Fig — (DOC) [file pone.0154287.s009.doc]

**S1 figure: Complete MMAT- VERSION 2011 from MMAT website http://mixedmethodsappraisaltoolpublic.pbworks.com. Archived by WebCite® at**

**http://www.webcitation.org/5tTRTc9yJ**

**
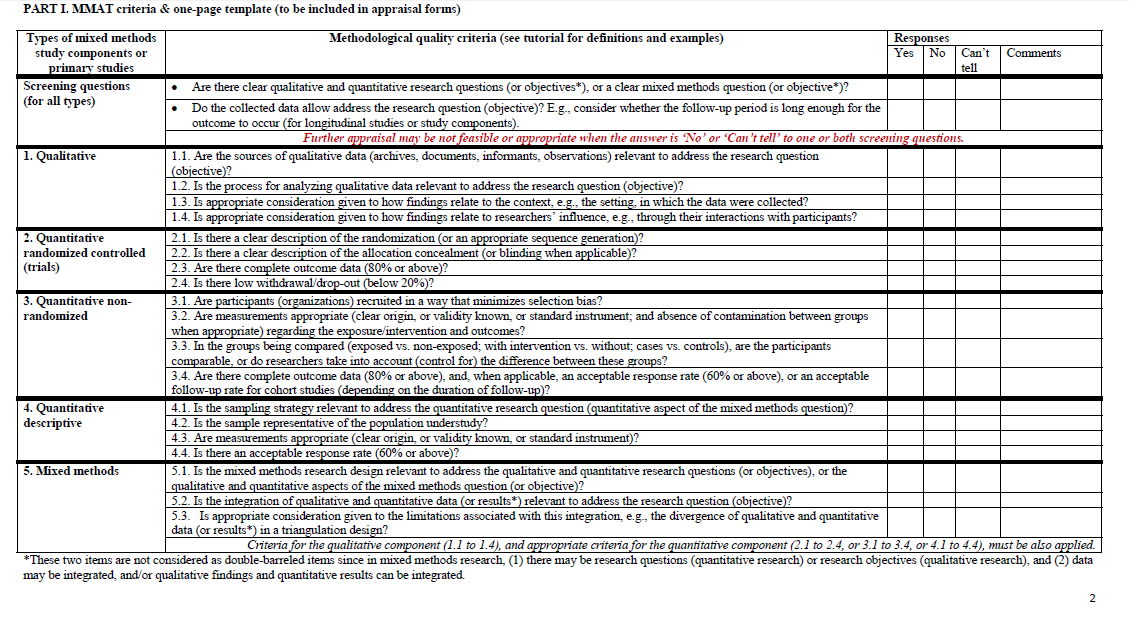
**
