# Supplementary material for: Beliefs of Health Care Providers, Lay Health Care Providers and Lay Persons in Nigeria Regarding Hypertension. A Systematic Mixed Studies Review
Source: PLoS One. 2016 May 5;11(5):e0154287. doi: 10.1371/journal.pone.0154287 (PMC4858295; doi:10.1371/journal.pone.0154287)
Supplement: S8 Table — (DOC) [file pone.0154287.s010.doc]

**S8 Table**: quality appraisal of quantitative studies

| **Study, Year** | **Study sample represents population of interest on key characteristics, sufficient to limit potential bias to results** | **Response rate (%)** | **Are measurements appropriate (clear origin, or validity known, or standard instrument)?** | **Is the sampling strategy relevant to address the quantitative research question** | **Controlled for confounding (if applicable)** |
| --- | --- | --- | --- | --- | --- |
| **Atulomah et al 2010** | YES | Not available | YES | YES | Not available |
| **Oke et al 2004** | YES | 68.3 | NO | YES | Not available |
| **Azubike et al, 2014,** | YES | 100 | YES | YES | Not available |
| **Familoni et al 2004** | YES | 100 | NO | YES | Not available |
| **Okwuonu et al 2014(a)** | YES | 64 | YES | YES | Not available |
| **Okwuonu et al 2014(b)** | YES | Not available | YES | YES | Not available |
| **Ike et al 2010** | YES | Not available | YES | YES | Not available |
| **Oladapo et al 2013** | YES | Not available | NO | YES | Not available |
| **Salaudeen et al 2014** | YES | 100 | NO | YES | Not available |
| **Adamu et al 2012** | YES | 100 | NO | YES | Not available |
| **Adeniyi et al 2015** | YES | 100 | YES | YES | Not available |
