# Supplementary material for: Beliefs of Health Care Providers, Lay Health Care Providers and Lay Persons in Nigeria Regarding Hypertension. A Systematic Mixed Studies Review
Source: PLoS One. 2016 May 5;11(5):e0154287. doi: 10.1371/journal.pone.0154287 (PMC4858295; doi:10.1371/journal.pone.0154287)
Supplement: S9 Table — (DOC) [file pone.0154287.s011.doc]

**S9 Table**: Examples of beliefs statements on hypertension per EM among qualitative studies

| **Explanatory Model** | **Some examples of Lay Healthcare providers belief statements** | **Some Examples of Lay Persons (hypertensive and non-hypertensive )beliefs statements** | **Some Examples of Health Care provider beliefs** |
| --- | --- | --- | --- |
| **Definition &**  **Causes** | - *"Hypertension is poor man's sickness. The stress in people's lives is too much; people are fighting and quarrelling with each other and placing cause on them or using witchcraft. All these are the cause of this disease that western medicine cannot cure and it is in our*  *blood and body"(CAM practitioner belief) (Osamor)*  *- "Hypertension is caused by too much blood in the body. That is why they call it high blood pressure sometimes. This causes the patient to always have headache and make the heart to beat too fast. It is this too much blood that sometimes make people to paralyze and unable to talk or walk. It is a serious illness*  *and only western medicine cannot treat it" (CAM practitioner belief) (Osamor)* | -“Eh…it’s high blood pressure but eh… eh… medical term for it is hypertension as I  Understand”(Taylor )  - “Yeah, thinking, if, if, if your brain is too loaded, with so many things, thinking of  it, it can cause it [HTN]”(Taylor)  - “I quite know that environment in Nigeria does not make it possible for somebody  to…of eh…our…somebody of our age to have normal pressure”(Taylor)  - hypertension is something that brings  death in different forms; one can be working and  suddenly fall down and slump or become paralyzed. It is called ‘kosibale okan‘in Yoruba meaning ‘there’s no peace of mind’.(Odusola)  - I don’t get hypertension before…one of my pickin [children] come die…so that’s  Why(Taylor) | *-‘Hypertension is not like many other sicknesses like malaria that is easy to manage and cure. You just take your medicine for a couple of days and you feel better.(Osamor)* |
| **Course and Symptoms** | -"Hypertension is curable. It is like every other sickness that you take medicine and you are cured, and it can reoccur later. For example, if you have malaria and you take iba medicine, it will go. Does that mean that person will not have malaria again? That is what I am saying. It is the same thing with hypertension. It can be cured"  - "I really do not know if we can cure hypertension because there are some people that have been taking English and traditional medicine for years and they are still taking it. They still come to us to complain and some are dead. So I do not know if the sickness is cured. We just give them medicine to help them reduce the sickness and make them feel better for sometime" | **-** No, it won’t come back by the Grace of God (Taylor)  - “First, I don’t fear about it because with God, all things are possible. I don’t fear about it. I know God can heal. I think you understand? I don’t…I don’t think am… much about it. But what I know is that eh…my drugs, I take it. When I get money I buy it and take it, not always oh. I don’t take it every time (Taylor) | **NA** |
| **Treatment** | *"Hypertension is curable. It is like every other sickness that you take medicine and you are cured, and it can reoccur later. For example, if you have malaria and you take iba medicine, it will go. Does that mean that person will not have malaria again? That is what I am saying. It is the same thing with hypertension. It can be*  *cured"(CAM practitioner belief) (Osamor)*  *- "I really do not know if we can cure hypertension because there are some people that have been taking English and traditional medicine for years and they are still taking it. They still come to us to complain and some are dead. So I do not know if the sickness is*  *cured. We just give them medicine to help them reduce the sickness and make them feel better for sometime"(CAM practitioner belief)*  *(Osamor)*  - *‘When I give them medicine, I ask them to go to the community health centre once in a while to check their blood pressure. I do not refer them. I treat them myself and some are well and cured.’ (CAM practitioner belief) (Osamor)*  - *Many of our customers come to us for help. In fact, from my records, I have about 12 customers that I am currently treating on hypertension. Majority of them come to us when they are sick and cannot afford to go*  *to UCH. They prefer coming to us because we are within the community and we are easy to reach"*.(Osamor CAM healer)  *-"Some of these people do not like going to the hospital because there is too much wahala [*too much trouble]*.It takes time to see the doctor and when they write drugs for them, it is not in the hospital and when they go out to buy, it is more expensive. Hence they sometimes come to us because we can treat on credit. No*  *hospital will take credit"*.(Osamor CAM healer) | **-** “…mix it together then we begin to drink, we call it Agbo in Yoruba area here. Yes…that means concoction…mixtures of some leaves, then all those things we mix it together. Then cook it, and then we drink it. A times it work, a times when it a very hard thing, then we turn to the orthodox doctors”(Taylor)  - can’t say... I can’t say all that… it’s the doctor that will say: this is the type of the drug that you are going to take…because I’m not a doctor (Taylor)  - “…mix it together then we begin to drink, we call it Agbo in Yoruba area here. Yes…that means concoction…mixtures of some leaves, then all those things we mix it together. Then cook it, then we drink it. A times it work, a times when it a very hard thing, then we turn to the orthodox doctors”(Taylor)  - apart from the pills given to me by my  doctor, I also take ‘bitter leaf water’; it is said to be  good for hypertension (Odusola)  - I am into herbs and I have used them severally in the past but they did not work like the white man’s medicine(Odusola)  - I use only the drugs prescribed to me, but ordinarily, as a member of Christ Apostolic  Church Christian faith, I really do not use drugs if not that this is really important; I believe in faith healing.(Odusola) | **NA** |

**NA=not available**
